# Standard Versus Ultrasound-Guided Cannulation of the Femoral Artery in Patients Undergoing Invasive Procedures: A Meta-Analysis of Randomized Controlled Trials

**DOI:** 10.3390/jcm9030677

**Published:** 2020-03-03

**Authors:** Sabato Sorrentino, Phong Nguyen, Nadia Salerno, Alberto Polimeni, Jolanda Sabatino, Angela Makris, Annemarie Hennessy, Gennaro Giustino, Carmen Spaccarotella, Annalisa Mongiardo, Salvatore De Rosa, Craig Juergens, Ciro Indolfi

**Affiliations:** 1 Division of Cardiology, Department of Medical and Surgical Sciences, Magna Graecia University, Viale Europa, 88100 Catanzaro, Italy; sabatosorrentino@hotmail.com (S.S.); polimeni@unicz.it (A.P.); jolesbt@hotmail.it (J.S.); c.spaccarotella@iol.it (C.S.); mongiardo@unicz.it (A.M.); saderosa@unicz.it (S.D.R.); 2 Campbelltown Hospital, Campbelltown, NSW 2560, Australia; ndtphong1@gmail.com (P.N.); An.Hennessy@westernsydney.edu.au (A.H.); 3 Liverpool Hospital, Liverpool, NSW 2170, Australia; a.makris@unsw.edu.au (A.M.); C.Juergens@unsw.edu.au (C.J.); 4 Western Sydney University, Campbelltown, NSW 2560, Australia; 5 University of New South Wales, Liverpool, NSW 1871, Australia; 6 Division of Cardiology, Ferrari Hospital, 87012 Castrovillari, Italia; nadia.salerno17@gmail.com; 7 Zena and Michael A. Wiener Cardiovascular Institute, Icahn School of Medicine at Mount Sinai, New York, NY 10029, USA; gennaro.giustino@muontsinai.org; 8 URT-CNR, Department of Medicine, Research Center for Cardiovascular Diseases, Viale Europa S/N, 88100 Catanzaro, Italy

**Keywords:** femoral artery, ultrasound, bleeding, vascular complications

## Abstract

Background: It is unclear whether or not ultrasound-guided cannulation (UGC) of the femoral artery is superior to the standard approach (SA) in reducing vascular complications and improving access success. Objective: We sought to compare procedural and clinical outcomes of femoral UGC versus SA in patients undergoing percutaneous cardiovascular intervention (PCvI). Methods: We searched EMBASE, MEDLINE, Scopus and web sources for randomized trials comparing UGC versus SA. We estimated risk ratio (RR) and standardized mean differences (SMDs) with 95% confidence intervals (CIs) for categorical and continuous variables, respectively. Primary efficacy endpoint was the success rate at the first attempt, while secondary efficacy endpoints were access time and number of attempts. Primary safety endpoints were the rates of vascular complications, while secondary endpoints were major bleeding, as well as access site hematoma, venepuncture, pseudoaneurysms and retroperitoneal hematoma. This meta-analysis has been registered on Centre for Open Science (OSF) (osf.io/fy82e). Results: Seven trials were included, randomizing 3180 patients to UGC (*n* = 1564) or SA (*n* = 1616). Efficacy between UGC and SA was the main metric assessed in most of the trials, in which one third of the enrolled patients underwent interventional procedures. The success rate of the first attempt was significantly higher with UGC compared to SA, (82.0% vs. 58.7%; RR: 1.36; 95% CI: 1.17 to 1.57; *p* < 0.0001; I^2^ = 88%). Time to access and number of attempts were significantly reduced with UGC compared to SA (SMD: −0.19; 95% CI: −0.28 to −0.10; *p* < 0.0001; I^2^ = 22%) and (SMD: −0.40; 95% CI: −0.58 to −0.21; p < 0.0001; I^2^ = 82%), respectively. Compared with SA, use of UGC was associated with a significant reduction in vascular complications (1.3% vs. 3.0%; RR: 0.48; CI 95%: 0.25 to 0.91; *p* = 0.02; I^2^ = 0%) and access-site hematoma (1.2% vs. 3.3%; RR: 0.41; CI 95%: 0.20 to 0.83; *p* = 0.01; I^2^ = 27%), but there were non-significant differences in major bleeding (0.7% vs. 1.4%; RR: 0.57; CI 95%: 0.24 to 1.32; *p* = 0.19; I^2^ = 0%). Rates of venepuncture were lower with UGC (3.6% vs. 12.1%; RR: 0.32; CI 95%: 0.20 to 0.52; *p* < 0.00001; I^2^ = 55%). Conclusion: This study, which included all available data to date, demonstrated that, compared to a standard approach, ultrasound-guided cannulation of the femoral artery is associated with lower access-related complications and higher efficacy rates. These results could be of great clinical relevance especially in the femoral cannulation of high risk patients.

## 1. Introduction

Access-site complications are the most common peri-procedural adverse event in patients undergoing trans-femoral percutaneous cardiovascular procedures and have been associated with an increased risk of morbidity, mortality and health-care costs [1,2]. Radial artery access is becoming the access of choice for the majority of diagnostic and coronary percutaneous interventions, thanks to its superficial and easily compressible nature, which ensures better hemostasis and lower rates of bleeding and vascular complications compared with the femoral route [3].

However, crossover from a planned trans-radial access to transfemoral access (TFA) still occurs in about 7% of cases, because of anatomical restrictions, including small artery size, the occurrence of radial artery spasm, or the presence of clinical conditions causing a poor radial pulse (i.e., cardiogenic shock or subclavian artery stenosis) [4]. Furthermore, procedures requiring the use of TFA due to large-diameter delivery sheaths, such as mechanical cardiovascular support, transcatheter valve replacement or aortic endoprosthesis implantation, are steadily increasing worldwide, further enlarging the number of patients requiring TFA [4,5]. 

Since success and freedom from complications are generally related to the operator case-volume, concerns have been raised about the fact that the new generation of interventional cardiologists, experienced primarily in the radial approach, are less skilled in performing procedures using TFA, a circumstance that potentially increases the risk of procedural complications [5,6]. In this scenario, adopting real-time ultrasound-guided cannulation (UGC) of the femoral artery may potentially decrease such adverse events. In recent years, several randomized controlled trials (RCTs) have been published evaluating the efficacy and safety of UGC in patients undergoing percutaneous cardiovascular interventions (PCvI), providing new insights within the context of new technologies and invasive procedures [7,8,9,10]. However, these trials were mostly underpowered to assess major vascular complications as a primary outcome, and the effect of UGC on clinically relevant vascular-access-related complications during femoral artery catheterization remains unclear. Therefore, ultrasound-guided cannulation is still not used routinely for vascular access in the cardiac catheterization laboratory, and is also not recommended in statements or current guidelines for interventional vascular procedures. 

Hence, we have undertaken a systematic review and meta-analysis evaluating the efficacy and safety of ultrasound-guided cannulation versus the standard approach in patients undergoing transcatheter diagnostic and interventional procedures using TFA.

## 2. Methods

### 2.1. Research Strategy and Study Design 

In accordance with PRISMA guidelines (Table S1 in the Data Supplement) [11], we searched MEDLINE, EMBASE, Scopus and oral presentations from the latest international conferences for papers published or posted until May 30, 2019. Our data were limited to randomized clinical trials (RCTs) enrolling patients undergoing invasive procedures using TFA. Exclusion criteria were observational studies, single arm pilot studies, non-English language studies, editorials, letters, expert opinions and case reports/series. The following key words were used for the search: femoral artery, ultrasonography, ultrasound, interventional, endovascular, cannulation, catheter, catheterization, angiography, percutaneous, and trial. Two investigators (S.S. and N.S.) independently evaluated studies for eligibility and any discrepancies were resolved by a third reviewer (A.P.). Risk of bias for each trial for both the primary and secondary endpoints were evaluated using the Cochrane tool, as described by Higgins et al. [12] The following potential sources of bias were evaluated: adequacy of random sequence generation (selection bias), allocation concealment (selection bias), blinding of participants and personnel (performance bias), blinding of outcome assessment (detection bias), description of incomplete outcome data (attrition bias) and selective reporting (reporting bias). For each element, a qualitative attribution of bias was given (low risk, intermediate risk, or high risk for bias) by two independent investigators (Table S2 in the Data Supplement). Data were independently extracted by two independent reviewers (S.S. and N.S.) and reported in a structured database. Discrepancies were resolved by a third reviewer. The primary efficacy outcome was the success rate at the first attempt. Secondary efficacy endpoints were access time and number of attempts. Primary safety endpoint was the rate of vascular complications, while secondary endpoints were major bleeding, access-site hematoma, pseudoaneurysm, venepuncture and retroperitoneal hematoma. All endpoints were assessed according to the definitions reported in the original trial protocols. 

### 2.2. Statistical Analysis

We estimated risk ratios (RRs) and standardized mean differences (SMDs) with 95% confidence intervals (CIs) for all available categorical and continuous variables, respectively, as previously described [13,14]. During data extraction, continuous variables, reported as medians with low and high ends of the range/interquartile range, were converted to means and standard deviations (SDs) according to the method of Wan et al. [15] Heterogeneity among trials for each outcome was estimated with chi-square tests and quantified with I^2^ statistics (less than 25% represented mild heterogeneity, 25%–50% represented moderate heterogeneity, and higher than 50% represented severe heterogeneity) [16]. The primary analytic method was random-effect models, according to Mantel–Haenszel. Publication bias was assessed by means of visual estimation of a Funnel plot. Study-specific influence on success rate at the first attempts and hematoma was estimated after the removal of each trial from the analysis and subsequent evaluation of the change in significance, magnitude, and direction of the effect. We deemed *p* values <0.05 as significant. RevMan (version 5.3) were used for the statistical analyses. This meta-analysis has been registered on PROSPERO (Registration Number: CRD42020150839).

## 3. Results

**Figure 1:**
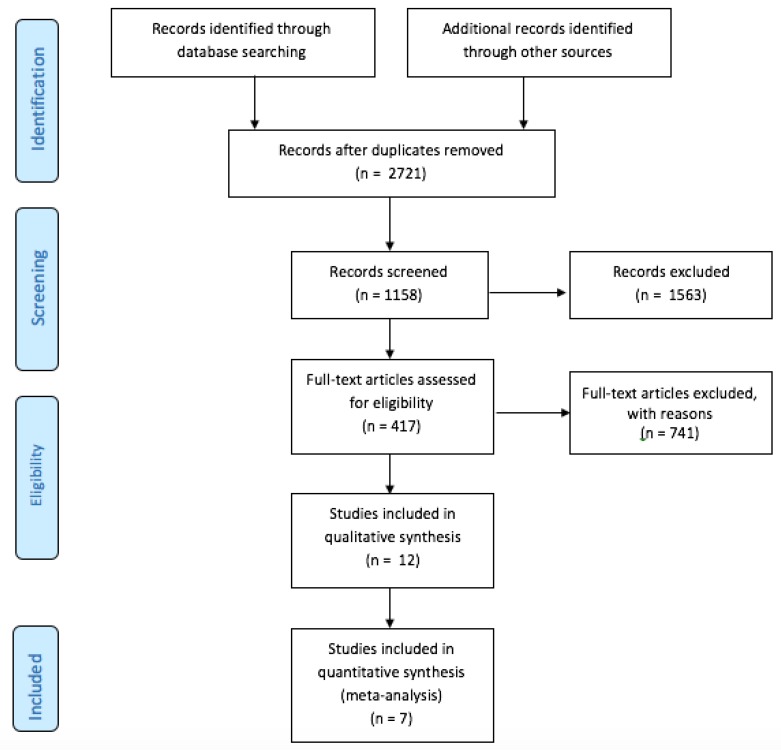
PRISMA Flow Diagram.

We included seven trials [7,8,9,10,17,18,19], in which 3180 patients were randomized to UGC (*n* = 1564) or SA (*n* = 1616). The detailed study flow diagram is shown in Figure 1. The main characteristics of each trial are reported in Table 1. Only one trial was powered to detect differences in vascular complications. Four trials reported the percentage of patients undergoing intervention procedures as almost one third of the enrolled patients. Procedures ranged from peripheral to coronary interventions.

**Figure 2:**
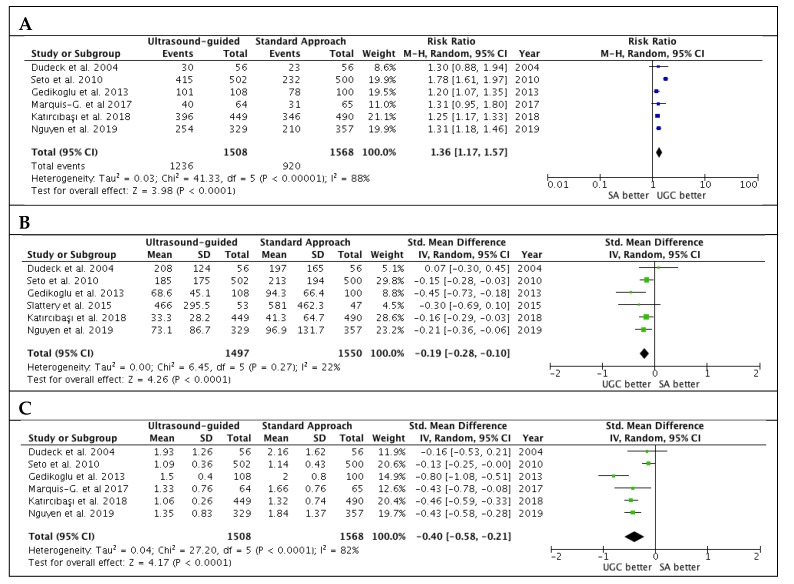
Estimates for success rate at the first attempts (**A**), time to access (**B**) and number of attempts (**C**) in patients undergoing femoral cannulation with or without ultrasound guidance. RR: risk ratio; CI: confidence interval; IV: inverse variance; SMD: standardized mean difference; UGC: ultrasound-guided cannulation; SA: standard approach.

**Figure 3:**
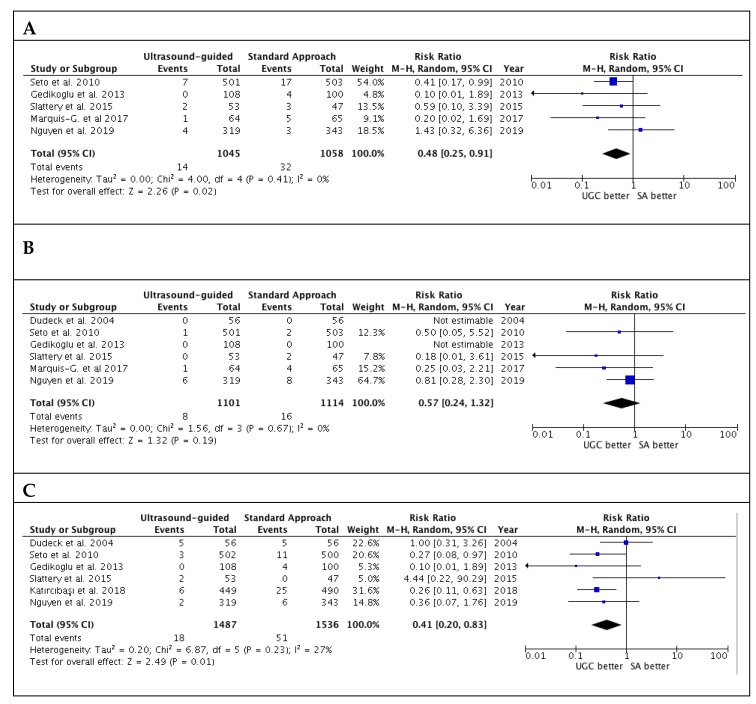
Risk estimates for vascular complications (**A**), major bleeding (**B**) and hematoma (**C**) in patients undergoing femoral cannulation with or without ultrasound guidance. RR: risk ratio; CI: confidence interval; UGC: ultrasound-guided cannulation; SA: standard approach.

Studies’ outcomes are summarized in Table 2. The success rate in the first attempts was significantly higher with UGC than SA, (82.0% versus 58.7%; RR: 1.36; 95% CI: 1.17 to 1.57; *p* < 0.0001; I^2^ = 88%) (Figure 2A). The magnitude and direction of the effect was consistent with the study influence analysis (Table S3 in the Data Supplement). Likewise, time to access in patients undergoing UGC was significantly lower compared to SA (SMD: −0.19; 95% CI: −0.28 to −0.10; *p* < 0.0001; I^2^ = 22%) (Figure 2B), as well as the number of attempts (SMD: −0.40; 95% CI: −0.58 to −0.21; *p* < 0.0001; I^2^ = 82%) (Figure 2C). No evidence of publication bias was observed (Supplementary Figure S1A–C). Patients randomized to UGC had lower rates of vascular complications compared to those who underwent TFA with SA (1.3% vs. 3.0%; RR: 0.48; CI 95%: 0.25 to 0.91; *p* = 0.02; I^2^ = 0%) (Figure 3A). The magnitude and direction of the effect was consistent with the study influence analysis (Table S4 in the Data Supplement). There were no significant differences between UGC and SA in terms of major bleeding complications (0.7% vs. 1.4%; RR: 0.57; CI 95%: 0.24 to 1.32; *p* = 0.19; I^2^ = 0%), but UGC was associated with a lower risk of access-site hematoma (1.2% vs. 3.3%; RR: 0.41; CI 95%: 0.20 to 0.83; *p* = 0.01; I^2^ = 27%) (Figure 3B–C). There was no evidence of publication bias for the risk of vascular complications, major bleeding, and access-site hematoma (Supplementary Figure S2A–C). 

**Figure 4:**
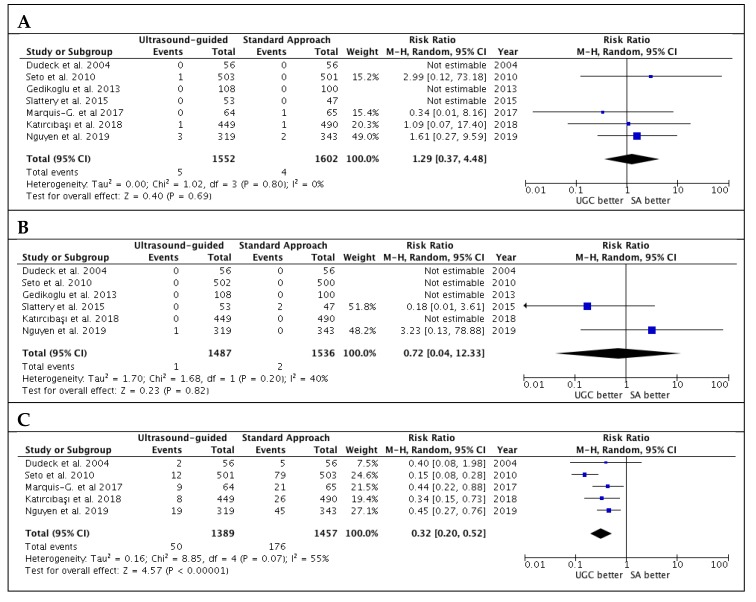
Risk estimates for pseudoaneurysm (**A**)**,** retroperitoneal hematoma (**B**) and venepuncture (**C**) in patients undergoing femoral cannulation with or without ultrasound guidance. RR: risk ratio; CI: confidence interval; UGC: ultrasound-guided cannulation; SA: standard approach.

Results for secondary safety endpoint are reported in Figure 4. There were no significant differences in terms of risk of pseudoaneurysm and retroperitoneal hematoma. However, UGC was associated with lower venepuncture rates (3.6% vs. 12.1%; RR: 0.32; CI 95%: 0.20 to 0.52; *p* < 0.00001; I^2^ = 55%). There was no evidence of publication bias for these outcomes (Supplementary Figure S3A–C).

## 4. Discussion

The main findings of the present meta-analysis comparing trans-femoral ultrasound-guided cannulation versus standard approach in patients undergoing PCvI are: (1) use of ultrasound-guided cannulation is associated with a higher rate of cannulation at the first attempt, a lower total number of attempts, as well as shorter time to access compared to SA; (2) patients undergoing trans-femoral ultrasound-guided cannulation of the femoral artery experienced a lower rate of vascular complications, including hematomas and venepuncture.

Based on the available evidence, UGC of the femoral artery appears to be superior to manual palpation or fluoroscopy guidance in decreasing the number of access failures and the overall time to sheath insertion. On the other hand, the studies published to date were not conclusive about risk reduction in vascular complications by UGC, because of their small sample size or the inclusion of low-risk patients. For instance, only two of the seven studies included in this analysis were designed to establish the impact of ultrasound guidance on clinical outcomes, studies moreover, that did not show a significant risk reduction in vascular complications with UGC.

The recently published standard versus ultrasound-guided radial and femoral access in coronary angiography and intervention (SURF) trial [7] is a factorial study design aiming to compare the effectiveness of trans-radial versus trans-femoral access and standard versus ultrasound-guided arterial access in patients undergoing cardiac catheterization. Of note, trans-radial access was superior to TFA in decreasing the rate of primary end-points including a composite acute catheterization and urgent intervention triage strategy (ACUITY) major bleeding, major adverse cardiac events (death, stroke, myocardial infarction or urgent target lesion revascularization) and vascular complications at 30 days (RR: 0.37, 95% CI: 0.17–0.81; *p* = 0.013), mostly driven by ACUITY major bleeding (RR: 0.343, 95% CI: 0.123–0.959; *p* = 0.041). However, UGC was not superior to the standard approach in terms of vascular complications, showing no differences in terms of hematoma >5 cm (RR: 0.72, 95% CI: 0.229–2.293; *p* = 0.58) and major bleeding (RR: 0.91, 95% CI: 0.369–2.263; *p* = 0.85) between groups. 

Conversely, in the large randomized trial published by Katırcıbaşı and colleagues [8], including a total of 939 patients undergoing a diagnostic or interventional coronary or peripheral procedure, the UGC group had a significant risk reduction for hematomas, and arteriovenous fistulas compared to the manual technique group. Likewise, Seto and colleagues [9], randomizing 1004 subjects undergoing fluoroscopic guidance versus real-time ultrasound cannulation of the femoral artery, showed a slightly lower rate of vascular complications in UGC patients compared to fluoroscopic guidance access. 

This heterogeneity in safety outcomes reported across the studies is mainly related to the different study designs, patients and procedural characteristics, as well as endpoint definitions, thus impairing comparability. For example, in the SURF trial, the absence of a significant difference between UGC and the control group may be related to the inclusion of patients undergoing radial approach, thus diluting the incidence rate of vascular complications, which is assumed to be significantly lower in this apporach. Nonetheless, the difference in vascular complications observed by Seto et al. may be chance, since this study was not powered for safety endpoints. 

In this meta-analysis, we summarized such evidence, documenting a lower rate of access site complications, hematomas and a non-significant 43% relative risk reduction (absolute risk reduction of 0.7%) in major bleeding events. Of note, such results are observed in patients undergoing, in most cases, relatively low-risk procedures with a standard TFA cannulation and smaller catheter (6 French), thus underlying a potential benefit in higher-risk procedures and patients. 

Preventing access site complications is a growing concern for several reasons. First, currently, radial is the access of choice for the majority of the percutaneous coronary procedures, and thus the younger generation of interventional cardiologists are less experienced in managing femoral cannulation and its related complications [5,6]. Second, an increasing number of TFAs are used for high-risk patients undergoing complex PCvI and for the implantation of left ventricular devices; percutaneous aortic valve and endoprosthesis implantation require transfemoral access for bigger devices [14,20,21,22]. Therefore, the magnitude of UGC benefits may potentially be greater in patients undergoing such high-risk interventions. Dudeck and colleagues have already reported such benefits in the UGC group versus SA, but only for obese patients or subjects presenting with a weak pulse [17]. Likewise, a single-center retrospective cohort study including 387 patients who underwent trans-femoral TAVR, showed that, compared to a standard access group, UGC-patients were less likely to experience access site complications and major bleeding events [23,24]. 

However, these findings were limited by either the small sample size or the retrospective design, thus requiring further investigation. Accordingly, the ongoing routine ultrasound guidance for vascular access for cardiac procedures (UNIVERSAL) trial (Clinical-Trials.gov Identifier NCT03537118), will provide new insights, enrolling 1538 patients to ultrasound-guided femoral artery access versus fluoroscopy, evaluating the rate of major vascular complications among patients referred for coronary angiography or percutaneous coronary intervention. 

This meta-analysis presents several limitations. First, most of the studies had a small sample size, and these results are mainly driven by the three largest RCTs [7,8,9] that formed almost 80% of the entire population. Second, outcomes and definitions varied among the studies and only three studies had blinded outcome assessments. Finally, we were unable to perform subgroup analyses based on procedural variables (access sheaths, type of procedures, use of closure devices, etc.), and clinical characteristics, such as obese patients or patients with other high-risk features. 

## 5. Conclusions

In conclusion, this meta-analysis, including seven RCTs, provides the most thorough analysis of ultrasound-guided cannulation of femoral artery for an endovascular procedure to date, confirming the superiority of UGC for efficacy endpoints as well as vascular complications, compared to standard or fluoroscopy guidance techniques. These results could have paramount importance in setting an internal hospital program of femoral cannulation according to the operator experience and the type of coronary or structural cardiac intervention.

## Figures and Tables

**Table 1:** Studies design and characteristics.

	Dudeck et al. 2004 [17]	Seto et al. 2010 [9]	Gedikoglu et al. 2013 [18]	Slattery et al. 2014 [19]	Marquis-Gravel et al. 2018 [10]	Katırcıbaşı et al. 2018 [8]	Nguyen et al. 2019 [7]
**Primary Endpoint**	No primary endpoints specified	CFA cannulation success	No primary endpoints specified	No primary endpoints specified	1-day immediate procedural outcomes and access-site outcomes *	No primary endpoints specified	30-day ACUITY major bleeding, MACE and vascular complications ^§^
**Sample Size**	112	1,004	208	100	129	939	688
**Randomization** **arms**	**UGC:** 56 **SA:** 56	**UGC:** 503 **SA:** 501	**UGC:** 108 **SA:** 100	**UGC:** 53 **SA:** 47	**UGC:** 64 **SA:** 65	**UGC:** 449 **SA:** 490	**UGC:** 331 **SA:** 357
**Comparator** **(SA)**	Arterial Palpation	Fluoroscopy guidance	Arterial palpation and Fluoroscopy guidance	Fluoroscopy guidance	Anatomical landmark and fluoroscopy as bail-out	Fluoroscopy guidance	Arterial palpation or Fluoroscopy guidance
**Age (mean, SD**)	**UGC:** 60 (15) **SA:** 60 (13)	**UGC:** 63.5 (12.4) **SA:** 64.2 (11.4)	**UGC:** 59 (15.2) **SA:** 59.5 (13.2)	**UGC:** 68 (49–92) **SA:** 66 (32–86) °	**UGC:** 65 (58–72) **SA:** 67 (59–72) ^**^	**UGC:** 60.3 (11.4) **SA:** 59.8 (10.6)	**UGC:** 63.2 (11.1) **SA:** 63.8 (11.3)
**Female**	**UGC:** 24 (42.9) **SA:** 18 (32.1)	**UGC:** 132 (26.2) **SA:** 135 (26.9)	**UGC:** 38 (35.2) **SA:** 34 (34.0)	**UGC:** 15 (28.3) **SA:** 16 (34.0)	**UGC:** 16 (25.0) **SA:** 18 (27.7)	**UGC:** 216 (48.1) **SA:** 233 (47.6)	**UGC:** 98 (29.6) **SA:** 103 (28.9)
**Interventional procedures**	NA	**USG:** 155 (30.8) **SA:** 161 (32.1)	NA	NA	**USG:** 36 (57.0) **SA:** 39 (62.0)	**USG:** 105 (23.5) **SA:** 90 (18.3)	**USG:** 89 (26.9) **SA:** 99(27.7)
**Sheath Sizes Used (French**)	4–5	5.6 (0.9) ^&^	5–7	NA	5–6	6	6–7
**UGC technique**	Short axis	Short axis	NA	NA	Short/Long axis	Short axis	Short/Long axis

Data reported as percentage (n/N) or mean ± standard deviation when appropriate. USG: ultrasound-guided; SA: standard approach; ACS: acute coronary syndrome; CFA: common femoral artery. * Access failure, ≥1 puncture attempts, transfixing arterial puncture, venepuncture, and catheter insertion outside of the CFA boundaries; Access-site outcomes: arteriovenous fistulae, pseudoaneurysm, dissections, thromboses, and significant bleeding; ^§^ ACUITY: Acute Catheterization and Urgent Intervention Triage strategy Y; MACE: major adverse cardiovascular events (death, stroke, myocardial infarction or urgent target lesion revascularization); Vascular complications: pseudoaneurysm, occluded radial artery, hematoma delaying discharge and deep vein thrombosis; NA: Not Available ^&^ data reported as continuous variable ± standard deviation, ° reported as range, ** reported as interquartile range.

**Table 2:** Outcomes in patients undergoing percutaneous cardiovascular procedures with and without USG.

	Dudeck et al. 2004 [17]		Seto et al. 2010 [9]		Gedikoglu et al. 2013 [18]		Slattery et al. 2014 [19]		Marquis-Gavel et al. 2018 [10]		Katırcıbaşı et al. 2018 [8]		Nguyen et al. 2019 [7]	
	USG	SA	USG	SA	USG	SA	USG	SA	USG	SA	USG	SA	USG	SA
**Efficacy endpoints**														
First-attempt success rate	30 (53.6)	23 (41.1)	415 (82.7)	232 (46.4)	101 (93.5)	78 (78.0)	NA	NA	40 (62)	31 (48)	396 (84)	346 (70)	254 (77.2)	210 (58.8)
Time-to-access (sec)	208 (124)	197 (165)	185 (175)	213 (194)	68.6 (45.1)	94.3 (66.4)	466 (295.5)	581 (462.3)	NA	NA	33.3 (28.2)	41.3 (64.7)	73.1 (86.7)	96.9 (131.7)
Number of attempts	1.93 (1.26)	2.16 (1.62)	1.3 (0.90)	3.0 (3.20)	1.5 (0.40)	2.0 (0.80)	NA	NA	1.33 (0.76)	1.66 (0.76)	1.06 (0.26)	1.32 (0.74)	1.35 (0.83)	1.84 (1.37)
**Safety endpoints**														
Vascular Complications	NA	NA	7 (1.4)	17 (3.4)	0 (0.0)	4 (4.0)	2 (3.8)	3 (6.4)	1 (2)	5 (8)	NA	NA	4 (1.3)	3 (0.9)
Hematoma	5 (8.9)	5 (8.9)	3 (0.6)	11 (2.2)	0 (0.0)	4 (4.0)	2 (3.8)	0 (0.0)	NA	NA	6 (1.3)	25 (5.1)	2 (0.6)	6 (1.8)
Major Bleeding *	0 (0.0)	0 (0.0)	1 (0.2)	2 (0.4)	0 (0.0)	0 (0.0)	0 (0.0)	2 (4.3)	1 (2)	4 (7)	NA	NA	6 (1.9)	8 (2.3)
Pseudoaneurysm	0 (0.0)	0 (0.0)	1 (0.2)	0 (0.0)	0 (0.0)	0 (0.0)	0 (0.0)	0 (0.0)	0 (0.0)	1 (2.0)	1 (0.2)	1 (0.2)	3 (0.9)	2 (0.6)
Retroperitoneal hematoma	0 (0.0)	0 (0.0)	0 (0.0)	0 (0.0)	0 (0.0)	0 (0.0)	0 (0.0)	2 (4.3)	NA	NA	0 (0.0)	0 (0.0)	1 (0.3)	0 (0.0)
Venepuncture	2 (3.6)	5 (8.9)	12 (2.4)	79 (15.8)	NA	NA	NA	NA	9 (14)	21 (32)	8 (1.7)	26 (5.3)	19 (5.8)	45 (12.6)

Data reported as percentage (n/N) or mean ± (standard deviation) when appropriate. USG: ultrasound-guided; SA: standard approach. * Major Bleeding: defined as retroperitoneal hemorrhage or major bleeding assessed using BARC or Acuity criteria; NA: not available.

## Supplementary Materials

The following are available online at https://www.mdpi.com/2077-0383/9/3/677/s1, Figure S1: Funnel Plot analysis for success rate at the first attempts (A), time to access (B) and number of attempts (C) in patients undergoing femoral cannulation with or without ultrasound guidance, Figure S2: Risk estimates for vascular complications (A), hematoma (B) and (C) major bleeding in patients undergoing femoral cannulation with or without ultrasound guidance, Figure S3: Funnel Plot analysis for pseudoaneurysm (A), retroperitoneal hematoma (B) and venepuncture (C) in patients undergoing femoral cannulation with or without ultrasound guidance. Table S1: PRISMA Checklist, Table S2: Risk of bias assessment by 7 Domains of the Cochrane Risk of Bias Tool, Table S3: Trial influential analysis for the primary efficacy endpoint (success rate at the first attempt), Table S4: Trial influential analysis for the primary safety endpoint (vascular complications).

## Abbreviations

PCvI	percutaneous cardiovascular interventions
UGC	ultrasound guided cannulation
SA	Standard approach
MB	Major Bleeding
TFA	Trans-femoral access
ACUITY	Acute Catheterization and Urgent Intervention Triage strategY
ACS	acute coronary syndrome
CFA	common femoral artery
MACE	major adverse cardiovascular events
